# Drying, a practical technology for reduction of poultry litter (environmental) pollution: methods and their effects on important parameters

**DOI:** 10.1016/j.psj.2024.104277

**Published:** 2024-08-30

**Authors:** Mehdi Khodadadi, Aminolah Masoumi, Morteza Sadeghi

**Affiliations:** Department of Biosystems Engineering, College of Agriculture, Isfahan University of Technology, Isfahan, 84156-83111, Iran

**Keywords:** ammonia emission, broiler litter, elemental content, laying hen manure, microbial load

## Abstract

Poultry farming generates significant poultry litter (broiler litter and laying hen manure), posing environmental and human health risks. Heat treatment, particularly through drying, can mitigate these adverse effects. This paper aimed to explore the impact of various drying methods of poultry litter on key process indicators. The literature review showed that the drying kinetics of the broiler litter in a hot air dryer is affected by the manure depth, air velocity, drying temperature, and air relative humidity. Nevertheless, the effect of the air relative humidity is insignificant on drying laying hen manure. Hot air drying, freeze drying, and oven drying have significant effects on the nutrient content of the broiler litter. In drying both broiler litter and laying hen manure, the specific energy consumption decreases as air temperature and relative humidity rise. Low temperatures cause poor bacteria destruction in poultry litter, but at temperatures over 50°C, *Salmonella* is completely destroyed. The ammonia release from laying hen manure and broiler litter is very sensitive to moisture content. Generally, air temperature, air velocity, manure depth, and air relative humidity positively correlate with ammonia emission. The average ammonia emission during belt drying of laying hen manure is about 209.3 mg NH_3_ d^-1^ hen^-1^. Finally, the lack of comprehensive research on poultry litter drying with modern drying methods (ultrasound, microwave, infrared rays, and freeze drying) is evident. One approach that may offer new opportunities is hybrid methods, such as a combination of dryers that use hot air drying agents with these modern drying methods.

## INTRODUCTION

One of the most efficient ways to provide protein sources and food security for different communities is poultry farming, which produces a large amount of poultry litter (broiler litter and laying hen manure) (Castro et al., 2023). The poultry litter is a problematic agricultural waste because its disposal and storage have become an environmental concern due to the associated water, soil, and air pollution (Bad odor, greenhouse gases, and emission of NH_3_, H_2_S and etc). Moreover, the poultry litter contains more concentrations of phosphorus (**P**), calcium (**Ca**), nitrogen (**N**), and many other components than other animal manures (such as K, Mg, Fe, Cu, Zn, Mn, B, P_2_O_5_, and K_2_O) (Aboltins and Kic, 2015; cha et al., 2020; Kacprzak et al., 2023). Broiler litter and laying hen manure constitute the main parts of the poultry litter in the poultry breeding industry. It has been reported that during a breeding period, broilers generate an average of 1.18 kg of litter per bird, and a laying house with 100,000 hens produces an average of 2000 tons of manure annually (Ashworth et al., 2020).

Nitrogenous compounds of the poultry litter (urea, undigested proteins, and ammonium) are the main sources of ammonia emission and bad odor caused by the anaerobic bacteria' activities in the litter. Conversion rate of these compounds to ammonium and finally ammonia gas is affected by moisture and temperature content of the poultry litter (Khodadadi et al., 2023a). Research indicates that the moisture content of the broiler litter is more than 50%, and that of the laying hen manure collected under the battery cage of a laying house is more than 70%. This high moisture can lead to faster reproduction of bacteria/pathogens and more ammonia emissions, while heating can reduce microbial load and ammonia emission (Ghaly and MacDonald, 2012a; Li et al., 2020a). The poultry litter may also release various greenhouse gases such as CO_2_, CH_4_, N_2_O, H_2_S, and volatile organic compounds depending on its moisture content and pH content. Also, with microbial processes of nitrification and denitrification, N_2_O is emitted from the poultry litter to the atmosphere and contributes to stratospheric ozone depletion (Li and Xin, 2010).

To reduce the mentioned adverse effects of the poultry litter on the environment, the broiler litter and laying hen manure should be treated, and one of the best methods is drying for better storage, easier transportation, costs reduction of logistics, and reduction of ammonia emission and environmental hazards (Aboltins and Kic, 2015; Khodadadi et al., 2023b). Drying can affect the pH and elemental content of the broiler litter and laying hen manure such as calcium, phosphorus, potassium, nitrogen and a plurality of micronutrients (Staroń et al., 2016).

Therefore, in order to better assist researchers in their future work, it will be very beneficial to investigate various drying techniques for laying hen manure and broiler litter and their impacts on drying kinetics and the elements composition of the poultry litter.

The goal of this review is to present some experimental and theoretical investigations on drying broiler litter and laying hen manure and compare some drying technologies that could be generalized. The scope of this review includes: (1) Characteristics of raw broiler litter and laying hen manure, (2) Different drying methods, (3) Drying kinetics of broiler litter and laying hen manure in each drying method, (4) Effect of each drying method on the changes of the elements in the broiler litter and laying hen manure, (5) Energy consumption during the drying process (6) Changes in microbial load of the broiler litter and laying hen, and (7) Ammonia emission during the drying process.

## CHARACTERISTICS OF RAW BROILER LITTER AND LAYING HEN MANURE

Table 1 compares the moisture content, pH, ammonium nitrogen, total Kjeldahl nitrogen, and some elements (Ca, N, P, and K) of raw broiler liter and laying hen manure. As can be seen, more characteristics of the broiler litter and laying hen manure differ.

**Table 1:** Some reported characteristics for broiler litter and laying hen manure.

Item	Laying hen manure		Broiler litter	
	Value	Reference	Value	Reference
Moisture content (%wb)	72.9	(Chepete et al., 2011)	58.6	(Khodadadi et al., 2023a)
	74.8	(Li et al., 2020a)	59.5	(Bhatnagar et al., 2020)
	76.4	(Li et al., 2020b)	53.2	(Dunlop et al., 2016)
	78.4	(Li et al., 2023)	63	(Fuchs et al., 2018)
pH	8.6	(Chepete et al., 2011)	6.37	(Khodadadi et al., 2023a)
	8.2	(Li et al., 2020a; b)	6.31	(Szogi et al., 2019)
	8.4	(Khademi et al., 2024)	6.1	(Singh et al., 2018)
	8.8	(Font-Palma, 2012)	6.47	(Toppel et al., 2019)
Ammonium nitrogen (g kg^−1^)	4.5	(Chepete et al., 2011)	14.4	(Shapovalov et al., 2020)
	6.9	(Zhang et al., 2020)	17.6	(Font-Palma, 2012)
	7.8	(Li and Xin, 2010)	23	(Wedwitschka et al., 2020)
Total Kjedahl nitrogen (g kg^−1^)	47.2	(Molaey et al., 2018)	55.2	(Singh et al., 2018)
	56.53	(Li et al., 2020b)	69	(Selvaraj et al., 2018)
	57.37	(Li et al., 2020a)	52.5	(Markou, 2015)
Ca (%)	7.6	(Chastain et al., 2010)	3.89	(Shapovalov et al., 2020)
	10.6	(Topcu et al., 2022)	1.98	(Vimal et al., 2022)
	6.86	(Løes, 2017)	2.6	(Wang et al., 2015)
P (%)	2.17	(Topcu et al., 2022)	5.62	(Kabelitz et al., 2021)
	1.2	(Fuchs et al., 2018)	3.6	(Bhuiyan et al., 2015)
	2.5	(Bhuiyan et al., 2015)	4.2	(Ghanim, 2018)
K (%)	2.38	(Quiroga et al., 2010)	2.09	(Shapovalov et al., 2020)
	2.38	(Fuchs et al., 2018)	3.14	(Jurgutis et al., 2020)
	1.3	(Bhuiyan et al., 2015)	2.45	(Vimal et al., 2022)
N (%)	3.39	(Fuchs et al., 2018)	8.43	(Isemin et al., 2017)
	5.9	(Quiroga et al., 2010)	8.5	(Ksheem et al., 2015)
	5.49	(Tańczuk et al., 2019)	8	(Huang et al., 2015)

***Moisture content*****:** As presented in Table 1, the moisture content reported by researchers for laying hen manure is higher than the moisture content reported for broiler litter. A mixture of chicken excrement and materials generated during the growth of broilers (such as soil, insects, feathers, leftover food, etc.) and some other materials (such as wood shavings, peanut hulls, pine straw, and other absorbent dry low-cost materials) that are spread as litter in the broiler houses and absorb water and incorporate manure is known as broiler litter (Toledo et al., 2019). Litter is not removed from the broiler houses during the breeding period of the broiler chickens (Kuleile et al., 2019). Therefore, by the end of the breeding period, the litter will lose some of its moisture, and if the broiler houses are equipped with floor heating, the moisture reduction will be much greater. Laying hen manure is without bedding material, and as a result, its dry matter content is low (Ara et al., 2008). Unlike the broiler houses, the manure in laying hen houses is typically scraped daily or several times a week from below the caged laying birds and collected in large storage piles. Consequently, the laying hen manure has less time to lose moisture, which is why the moisture content of the laying hen manure is higher than that of the broiler litter (Liang et al., 2005a).

***pH*****:** Poultry litter pH is the main factor in the biological and chemical decomposition of its organic and inorganic matter and plays a vital role in NH_3_ emission from the poultry litter (Li and Ni, 2021). Table 1 shows that the pH of the laying hen manure is higher than that of the broiler litter. The diet of the laying hens contains more sources of calcium than that of the broilers to promote appropriate eggshell development (Kakhki et al., 2019). The primary source of calcium used in the diet of the laying hens is calcium carbonate or ground lime. Therefore, the calcium carbonate in feed waste and its excretion through feces is the reason for the higher pH of the laying hen manure than the broiler litter. Therefore, the laying hen manure can be used to improve acidic soils (Judge, 2001).

***Ammonium nitrogen*****:** Ammonium nitrogen (**NH_3_-N**) is one of the most significant sources of the poultry litter nitrogen losses to the atmosphere (Liang et al., 2005b). Usually, laboratories report a single value for the ammonium nitrogen content of the poultry litter, which includes different forms of ammoniacal nitrogen (${\text{NH}}_{4}^{+}$ and NH_3_-N). In most cases, the combination of NH_3_-N and ${\text{NH}}_{4}^{+}$ is known as total ammoniacal nitrogen (**TAN**). TAN and Total Kjeldahl nitrogen (**TKN**) can be obtained using laboratory analyses, and organic nitrogen is obtained by subtracting TAN from TKN (Ashworth et al., 2020). In general, the poultry litter has more ammonium nitrogen content than other manures. About 70% to 80% of nitrogen content in the poultry litter is in organic form and the rest of it is in ammonium form. The data in Table 1 show that the ammonium nitrogen content in the broiler litter is much higher than in the laying hen manure. Most of the nitrogen in the poultry litter is derived from protein (Wood et al., 2010). Because more protein is used in the diet of the broilers than that of the laying hens, the excretion of undigested proteins, urea, and uric acid is also higher by the broilers. This explains the significant difference between ammonium nitrogen in the broiler litter and laying hen manures.

***Total Kjeldahl Nitrogen (TKN)*****:** As seen in Table 1, the TKN content of the broiler litter and laying hen manure is very close. Because the ammonium nitrogen of the broiler litter is significantly higher than that of the laying hens, the organic nitrogen of the broiler litter is less than that of the laying hens, and its inorganic nitrogen is more than that of the laying hens. Organic nitrogen is unavailable to plants until converted to inorganic nitrogen (ammonium nitrogen) (Ashworth et al., 2020). As a result, the nitrogen content of the broiler litter is better available to plants.

***Calcium*****:** Calcium is one of the main ingredients in the diet of the laying hens for the production and optimum quality of eggshells (An et al., 2016). During the laying period, hens excrete a lot of calcium with eggs (20–30 times more than the total calcium reserves in the chicken's body). Therefore, to compensate for that, the laying hen's diet should contain more calcium. (David et al., 2023). This makes the laying hens excrete more calcium in the feces. As can be seen in Table 1, the calcium content of the laying hen manure reported in various research is much higher than that of the broiler litter.

***Phosphorus*****:** Manure is rich in nutrients, including trace elements such as phosphorus(P) necessary for plant growth. Approximately 60% to 85% of phosphorus in the diet is excreted through manure (Herbert et al., 2020). phosphorus in poultry litter is present mainly in the solid-phase as organic and inorganic Phosphorus. According to the literature (Table 1), broiler litter contains more phosphorus than laying hen manure. Also, the percentage of available phosphorus in broiler litter is higher than that of laying hen manure (Kacprzak et al., 2023). Dróżdż et al. (2023) reported that mixing poultry manure with litter will increase its phosphorus content, so it seems reasonable that the phosphorus content in broiler litter is higher than that of laying hen manure. Because phosphorus occurs in the form of inviable phytin in barley, soybean, wheat bran, and rapeseed meal, the phytic form is present in significant amounts in poultry litter (Ashworth et al., 2020). Available phosphorus sources for birds are mineral supplements added to feed. The range of 32–84% wt for inorganic phosphorus and 14% to 68% wt for organic phosphorus in poultry litter indicates the possibility of mineralization into a plant-available form (It is believed that 80% of the phosphorus in manure will be accessible to crops) (He et al., 2008). The solid phase of phosphorus is not completely organic but is a combination of minerals and organic compounds. The inorganic phosphate fraction contains dibasic calcium phosphate, calcium phosphate, and poorly bound water-soluble phosphorus. Both portions of total phosphorus are essentially immediately labile, but phytates are an exception (Kacprzak et al., 2023).

***Potassium*****:** Approximately 60% to 85% of potassium (**K**) in the diet is excreted through manure (Herbert et al., 2020). As presented in Table 1, broiler litter and laying hen manure do not differ much in potassium content. Potassium as a primary intracellular cation has dietary alkalogenic effect. It has been reported that the presence of potassium in the diet increases the moisture content of the poultry litter because the use of potassium in the diet leads to increased water consumption and litter moisture (Koreleski et al., 2010). But some researchers believe that using potassium in the diet reduces the moisture content of the laying hen manure. Joardar (2019) reported that adding 0.2% potassium to the diet reduces the moisture content of the laying hen manure.

***Nitrogen*:** The reported results show that the nitrogen content of the broiler litter is higher than that of the laying hen manure (Table 1). The higher protein content in the diet of the broilers may be the reason (Ji et al., 2014). In the broiler breeding industry, chickens use more nutrients, so they excrete denser nutrients. It has been reported that approximately 70%-80% of the nitrogen (N) in the diet is excreted through manure (Herbert et al., 2020).

## POULTRY LITTER DRYING METHODS

**Figure 1:**
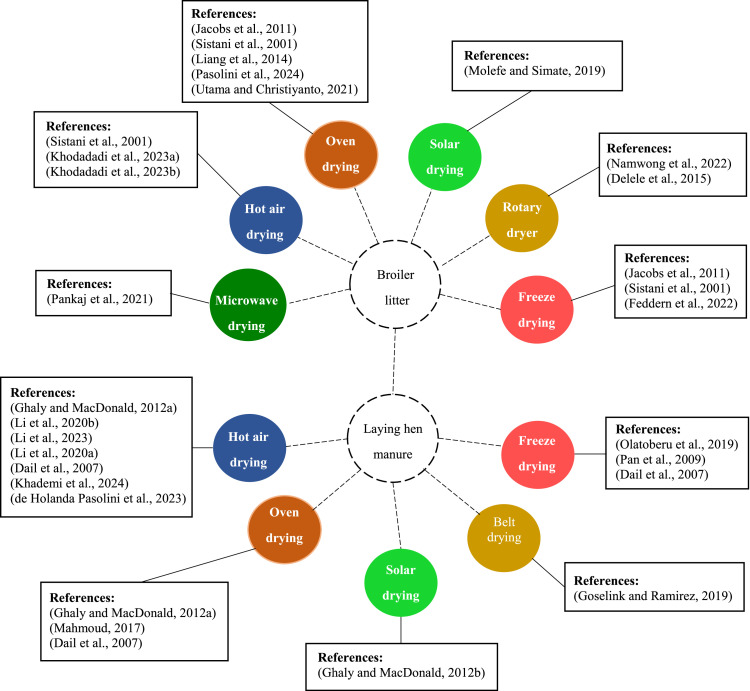
Application of different drying methods for broiler litter and laying hen manure (Delele et al., 2015, Feddern et al., 2022, Goselink and Ramirez, 2019, Namwong et al., 2022, Olatoberu et al., 2019, Pan et al., 2009, Utama and Christiyanto, 2021).

As mentioned, drying is one of the best methods to reduce the adverse effects of the poultry litter on the environment. Drying is a thermal treatment whose basic objective is to remove the water content to produce a solid product (reduce water activity) and prevent microbial spoilage and various chemical reactions (Vargas et al., 2022). Depending on the applied heat transfer method, there are different types and designs of dryers. In most drying methods, heat is first transferred to the surface of the wet material and then to its interior. However, in radio frequency (**RF**), dielectric, and microwave drying, thermal energy is first generated inside the wet material and then flows to the outer surfaces (Mali and Butale, 2019). Poultry litter is hygroscopic, so its drying rate that is the amount of water removed per unit time is very critical. To increase the quality of the dried poultry litter and improve the drying efficiency, various drying techniques have been developed. Figure 1 shows the different methods that have been used by researchers to dry broiler litter and laying hen manure.

## POULTRY LITTER DRYING KINETICS

Predicting the drying kinetics of the poultry litter under different conditions is very important for energy management, process and equipment design, choosing suitable storage, and material transfer (Salamat et al., 2022). Drying dramatically affects the quality of the dried product. For this reason and to achieve a stable drying process, the selection of optimal operating conditions has always been taken into account by researchers. The type of dryer, drying conditions, and properties of the material to be dried all affect drying kinetics (Onwude et al., 2016). In this section, the drying kinetics of the broiler litter and laying hen manure in different dryers are discussed.

### Broiler Litter Drying Kinetics

As presented in Figure 1, many research have been carried out on drying broiler litter, but few of them have investigated the drying kinetics of the broiler litter. Khodadadi et al. (2023a) studied the hot air drying process of the broiler litter to investigate the process impact on ammonia emission from the material. They reported that the drying kinetics is affected by the depth of the broiler litter, drying temperature, and air velocity. Moisture loss increased with increasing air velocity and drying temperature as well as decreasing broiler litter depth. High relative humidity of the drying air slows down the drying rate, due to the fact that a high relative humidity lowers the moisture gradient between the broiler litter and the drying air, thereby increasing moisture diffusion. On the other hand, elevated relative humidity may cause the temperature of the broiler litter samples to rise quickly, and consequently apply a higher driving force for water diffusion. This can lessen the negative impact that high relative humidity has on the rate of drying. Broiler litter depth, air temperature, air relative humidity, and air velocity had the greatest effect on the drying rate of broiler litter, respectively.

Thin layer drying kinetics of broiler litter in a direct sun dryer were studied by Molefe and Simate (2019b). The authors reported that solar radiation from 220 to 1005 W/m^2^ resulted in an air temperature increase of up to 64°C in the collector, up to 60°C in the drying unit, and an ambient temperature increase of up to 31°C. Exposure of broiler litter with an average initial moisture content of 61% (w.b.) to these conditions led to final moisture content of 0.2% to 11.2% (w.b.) in 31 to 35 h. In addition, they fitted the moisture rate results in 12 models shown in Table 2. Among all models, the Logarithmic drying model was the most accurate in estimating how the broiler litter will dry. This model had coefficient of determination (**R^2^**) ranging from 0.993 to 0.999, chi-square (χ2) from 6.496E^-14^ to 1.3626E^-11^ and root mean square error (**RMSE**) from 0.0130 to 0.0294.

**Table 2:** Thin layer drying models (Molefe and Simate, 2019).

Model name	Model equation
Newton	MR= $e^{\left(-kt\right)}$
Page	*MR=*$\;e^{\left(-kt^{n}\right)}$
Modified Page	*MR=*$\;e^{{\left(-kt\right)}^{n}}$
Henderson and Pabis	*MR= a*$\;e^{\left(-kt\right)}$
Logarithmic	*MR= a*$e^{\left(-kt\right)}$*+c*
Two term	*MR= a*$e^{\left(-k_{0}t\right)}$*+b*$e^{\left(-k_{1}t\right)}$
Approximation of Diffusion	*MR= a*$e^{\left(-kt\right)}$*+(1-a)*$e^{\left(-kbt\right)}$
Verma et al.	*MR= a*$e^{\left(-kt\right)}$*+(1-a)*$e^{\left(-gt\right)}$
Modified Henderson and Pabis	*MR= a*$e^{\left(-kt\right)}$*+b*$e^{\left(-gt\right)}$*+c b*$e^{\left(-ht\right)}$
Two term exponential	*MR= a*$e^{\left(-kt\right)}$*+(1-a)*$e^{\left(-kat\right)}$
Wang and Singh	*MR= 1+at+b*$bt^{2}$
Midilli-kucuk	*MR= a*$e^{\left(-kt^{n}\right)}+bt$

Liang et al. (2014) investigated 4 treatments for drying of broiler litter: flask-drying at 70 °C for 16 h; flask-drying at 50°C for 24 h; oven-drying at 70°C, and oven-drying at 105°C to reach a constant mass. They indicated that moisture loss was significantly higher for the flask-drying at 70°C in comparison with 50°C. Drying was completed with the flask-drying at 70°C but not at 50°C. Therefore, flask-drying was as effective as forced-air drying in the oven at 70°C for 16 h.

### Laying Hen Manure Drying Kinetics

Pasolini et al. (2024) dried laying hen manure in a convective dryer with a temperature of 60, 70, and 80°C and air velocity of 1, 1.2, and 1.8 m s^-1^. Investigation of drying kinetics showed that moisture losses during the drying process were associated with higher air temperature. Nevertheless, the mechanisms of diffusive mass transfer and morphological characteristics of the laying hen manure cause low water evaporation rates. Effective diffusion and activation energy ranged from 2 × 10^-8^ to 9 × 10^-8^ m^2^ s^-1^ and from 45 to 67 kJ mol^-1^, respectively. Air velocity, and particularly temperature, influenced these parameters.

Khademi et al. (2024) optimized laying hen manure drying in a hot air dryer. They dried laying hen manure in 2 sets of experiments (drying with and without sodium bentonite (SB) and wheat straw (WS)). The independent parameters were air temperature (70, 80, and 90°C), air velocity (1, 1.5, and 2 m s^-1^), layer thickness (5, 10, and 15 mm), SB (2%, 4%, and 6%), and WS (3%, 7.5%, and 12%). According to their findings, the maximum rate of dehydration occurred at the lowest manure depth and the highest air velocity. Minimum drying duration occurred at an air temperature of 78°C, an air velocity of 1 m s^-1^, and a manure depth of 5 mm in the first set of experiments, and at an air temperature of 80°C, an air velocity of 1.5 m s^-1^, a layer thickness of 11 mm, 6% SB, and 12% WS in the second set of experiments. They concluded that adding wheat straw and sodium bentonite to laying hen manure can significantly reduce the drying time (temperature and WS had the most significant influence on dehydration rate).

In a humid environment Li et al. (2023) studied the effects of air velocity, drying air temperature, and thickness of manure on low temperature drying characteristics of laying hen manure. Their findings demonstrated that the drying process of the laying hen manure at low temperatures in a humid environment had only a falling rate period and no constant rate or accelerated period. In addition, the exponential model was considered more appropriate to describe the drying process. The effective diffusivity also altered between 6.37 × 10^-7^ and 2.17 × 10^-6^ m^2^ h^-1^. Moreover, they used Lewis, Page, Wang and Singh, Henderson and Pabis, and Exponential models to simulate the drying process' performance. The exponential model was shown to be the most effective in explaining the process of laying hen manure drying at low temperatures among all drying models.

de Holanda Pasolini et al. (2023) studied the chemical and physical properties and the effect of different operational conditions (air velocities of 1 and 1.4 m s^-1^ and air temperatures of 60 and 80°C) on drying laying hen manure. Based on their results, the drying kinetics and thermal efficiency were highlighted at 80°C and 1 m s^-1^ and 80°C and 1.4 m s^-1^. The effect of the temperature was more significant than the air velocity because an increase in temperature facilitated the mass transfer and as a result increased the moisture evaporation. They also fitted the moisture rate results in Page, Page Modified, Henderson Pabis, Lewis, Logarithmic, and Two Term models. The results showed that the Page and modified Page models were the best ones for predicting the drying behavior of the laying hen manure.

Lewis (2021) reported that the air relative humidity had no significant impact on drying laying hen manure, but Khodadadi et al. (2023b) reported that high air relative humidity values slowed down the drying rate of the broiler litter. This may be because the broiler litter contains materials generated during broiler production and some spread as litter in the broiler houses. Therefore, the difference in the ingredients of the broiler manure and laying hen manure could cause this difference.

Li et al. (2020b) looked at how drying laying hen manure was affected by temperature (15–35°C), air velocity (0.6–1.8 m s^–1^), and manure layer depth (60–140 mm). Their results indicated that the drying kinetics affected by the drying temperature significantly. An increase in air velocity and temperature led to an increase in dehydration rate, and the factor that most significantly affected how quickly the laying hen manure dried was the air temperature. The optimal parameters were as follows: hot air temperature of 35°C, air velocity of 1.60m/s, and manure layer thickness of 85 mm.

Mahmoud (2017) studied the effect of the air temperature (40, 50, and 60°C) and laying hen manure depth (1, 2, and 3 cm) on drying duration in a forced draft oven. The outcomes demonstrated that the thinner the depth of the manure, the shorter was the time required for moisture removal. Drying laying hen manure with a depth of 2 cm at 40, 50, and 60°C required 106%, 100%, and 87% more time compared to a depth of 1 cm, respectively. Drying laying hen manure with a depth of 3 cm at 40, 50, and 60°C also required 106%, 100%, and 87% more time versus a depth of 2 cm, respectively.

Aboltins and Kic (2015) investigated moisture reduction from laying hen manure in a forced convective hot air dryer where the air flow (1.13 m s^-1^ and 2.05 m s^-1^) was applied from the bottom to trays containing the manure with different holes’ sizes (3, 4, 5 mm). They stated in their report that the air flow had a significant efficacy on drying duration, while the size and shape of the holes were not crucial. Moreover, free convection was not practical for drying laying hen manure.

Ghaly and MacDonald (2012b) studied on drying laying hen manure in a solar dryer (manure depths of 1, 2, and 3 cm and temperatures of 40, 55, and 60°C). Their results showed that with increasing the drying temperature, the difference in drying duration between shallow and deeper layers decreased. In addition, in terms of drying effectiveness, the manure layer with a depth of 3 cm was superior at all temperature levels, because it took less time to extract 1 gram of water from the laying hen manure. They also reported that at all temperature levels, the time needed to extract 1 gram of water from the laying hen manure at a depth of 3 cm was the highest compared to other depths.

According to the presented literature, it can be seen that in various research, the focus has been on investigating the drying kinetics of the poultry litter in hot air dryers, oven dryers, and solar dryers, indicating that other dryers have been overlooked. Therefore, investigating the kinetics of poultry litter drying in other dryers to achieve the appropriate moisture content of poultry litter for subsequent applications, is necessary and should be considered by researchers in future research. Future research should expand upon the existing understanding of the drying process by investigating additional factors, as partially represented by the drying coefficient.

## CHANGES IN ELEMENTAL CONTENT DURING POULTRY LITTER DRYING PROCESS

Poultry litter contains higher elemental content than other animal manures. Most of these elements come from the birds' feed, supplements, medicines, and water. Also, the moisture and nutrient content of the poultry litter can be different depending on the type of poultry. Hence, different drying methods can have different effects on the amount of the poultry litter elements. In this section, the impact of drying method on the elemental changes of the broiler litter and laying hen manure has been discussed.

### Broiler Litter Elemental Content

Khodadadi et al. (2023b) studied the effect of the air relative humidity (8%–18%), air temperature (60–80°C), air velocity (2–3 m s^-1^), and broiler litter depth (2–4 cm) on crude protein changes of broiler litter during drying in a hot air dryer. According to their results, crude protein decreased with decreasing temperature, humidity, and air velocity. Also, changes in crude protein were most significantly impacted by air velocity. Low temperature led to longer drying duration, which in turn the crude protein could be further degraded or converted. Also, high temperatures can deactivate enzymes and prevent the breakdown of proteins. Therefore, drying poultry litter at high temperatures can result in less loss of crude protein during the drying process.

Pankaj et al. (2021) investigated on the changes in dielectric properties of broiler litter in the microwave drying process. In the current research, the dielectric properties of the broiler litter were measured at a frequency range of 10 MHz to 14 GHz with changes in moisture and temperature in the range of 15% to 40% (w.b.) and 10 to 70°C, respectively. They observed that the dielectric constant and dielectric loss coefficient decreased with increasing frequency at all moisture contents and temperatures. However, an increasing trend was observed with increasing moisture and temperature for the whole examined frequency range.

Jacobs et al. (2011) evaluated the impact of freeze drying and oven drying at 55°C and 100°C on nutrient concentrations in broiler litter. Their results showed that during the drying process, nitrogen (*P* < 0.10) and sulfur (*P* < 0.01) were decreased by an average of 10% and 66%, respectively. There were no considerable differences (*P* > 0.50) among drying methods except freeze drying broiler litter had a greater concentration of sulfur than oven drying (*P* < 0.10). without considering of drying method, some nitrogen loss appears to be unavoidable, but there is no obvious advantage between freeze drying and oven drying. Further sulfur loss warrants further investigation.

Fresh broiler litter was dried by López-Mosquera et al. (2008) using a drying tunnel set at 250°C. They observed that the drying process reduced variability in electrical conductivity, urea N, and K, dry matter content, Na, S, Fe, Cd, and Cu contents, but nevertheless increased variability in ammonium N, total N, total P, nitrate N, and pH. Compared to fresh broiler litter, the dried litter contained significantly lower levels of Cr, Cu and Cd. However, Pb content were notably higher in the dried litter. While the dried broiler litter offers distinct advantages in terms of storage and handling, the study indicates a need to refine the drying process to minimize fluctuations in nitrogen forms, phosphorus content, and pH.

Sistani et al. (2001) examined the impact of freeze drying, hot air drying, oven drying at 65°C, and 10 °C on broiler litter nutrient content. It was revealed that each drying method had a significant effect on the broiler litter's nutritional content. Total N decreased significantly at the end of all drying methods, but there was no discernible variation in total N losses among drying methods. The highest losses of calcium (**Ca**), phosphorus (**P**), magnesium (**Mg**), iron (**Fe**), manganese (**Mn**), potassium (**K**), and zinc (**Zn**) were related to the freeze drying. The effect of drying method on total N, organic N, and NH_4_-N was more severe than that of other nutrients. However, all drying methods resulted in 21% to 27% nitrogen. The pH changes due to drying were less than 1 unit, except for the oven drying method at 105 °C for which the pH was significantly lower than that of fresh broiler manure.

### Laying Hen Manure Elemental Content

Khademi et al. (2024) optimized the laying hen manure's drying procedure. They investigated the effect of wheat straw and sodium bentonite utilization on reducing crude protein and ammonium-N during the drying with a hot air dryer. The independent parameters were air velocity (1, 1.5, and 2 m s^-1^), air temperature (70, 80, and 90°C), manure depth (5, 10, and 15 mm), wheat straw (3%, 7.5%, and 12%), and sodium bentonite (2%, 4%, and 6%). Under the optimal conditions, the laying hen manure treated with 6% sodium bentonite and 12% wheat straw retained 10% more crude protein and 58% more ammonium-N than the untreated laying hen manure. During the drying of treated and untreated laying hen manure, when air velocity and air temperature increased in the experimental range, crude protein reduction increased. At the same time, manure depth, wheat straw, and sodium bentonite had the opposite effect. Compared to raw laying hen manure, treated samples had a significantly higher organic carbon content. This is due to the presence of a carbon source that prevents microorganisms from breaking down organic nitrogen. Microbes use wheat straw as a carbon source when added. For this reason, the reduction of crude protein decreased with the increase of the additive percentage. By increasing wheat straw and sodium bentonite additives, the rate of ammonium-N losses decreased. The reason is that wheat straw and sodium bentonite have a large surface area that can absorb ammonium N and prevent it from converting to ammonia gas. In laying hen manure drying without additives, increasing air velocity and air temperature in the experimental range caused more losses of ammonium-N, while manure depth had the opposite effect. This might be because more water is removed as a result of the temperature and air velocity increases, which removes more ammonium-N from the laying hen manure because of the release of ammonia.

Li et al. (2020a) looked at how the ammonium nitrogen (NH_4_-N) and organic nitrogen content changed over time when the laying hen manure dried at low temperatures. The drying experiments were conducted using 3 hot air temperatures: 15, 25, and 35°C. The relative humidity was maintained at 30% to 35% for all temperatures. The manure samples were spread to a thickness of 100 millimeters, and the air velocity beneath the samples was consistently 1.2 m s-1. Their findings demonstrated that NH_4_-N losses can rise with drying temperature. The organic nitrogen (**Org-N**) content in the laying hen manure did not vary significantly between the 3 drying temperatures used, even within the same drying time. This suggests that raising the temperature during the low-temperature drying process did not accelerate the breakdown of organic nitrogen.

Li et al. (2020b) investigated the nitrogen loss rate of laying hen manure during the drying process (air temperature of 15–35°C, air velocity of 0.6–1.8 m s^-1^ and manure layer thickness of 60–140 mm). They stated that with the increase of manure depth, nitrogen losses rate increased. The manure depth showed the most significant effect on the amount of nitrogen losses. The nitrogen loss was not positively correlated with the air temperature and air velocity.

Mahmoud (2017) studied the effects of the manure depth (1, 2, and 3 cm) and drying temperature (40, 50, and 60°C) on the elemental content of the laying hen manure in a forced draft oven. Their results showed that all amino acid concentrations in laying manure decreased during the drying process. The amino acid concentration decreased as the temperature increased and the depth of the manure decreased. The highest amounts of amino acids were detected at the lowest temperature and the deepest manure depth. The protein content of the dried laying hen manure was slightly lower than that of raw laying hen manure, but this difference was insignificant. It does not seem that the manure depth and drying temperature have any considerable effect on the final protein content. The investigated parameters had no significant impact on the concentration of phosphorus, potassium, and calcium but about 50% of nitrogen content was lost during the drying process.

Gunes et al. (2015) exposed the laying hen manure to air temperatures of 250, 300, 350, and 400°C to produce biochar. Their results indicated that high temperatures caused the mineral elements oxidation, breaking of C-H and C-C bonds, and removal of peptide and aliphatic groups from the pyrolyzed materials. The total concentrations of calcium (Ca), phosphorus (P), potassium (K), magnesium (Mg), iron (Fe), copper (Cu), zinc (Zn), manganese (Mn), and boron (B) were increased by increasing temperatures. However, except K and B, the water-soluble concentration of these elements was significantly reduced.

**Figure 2:**
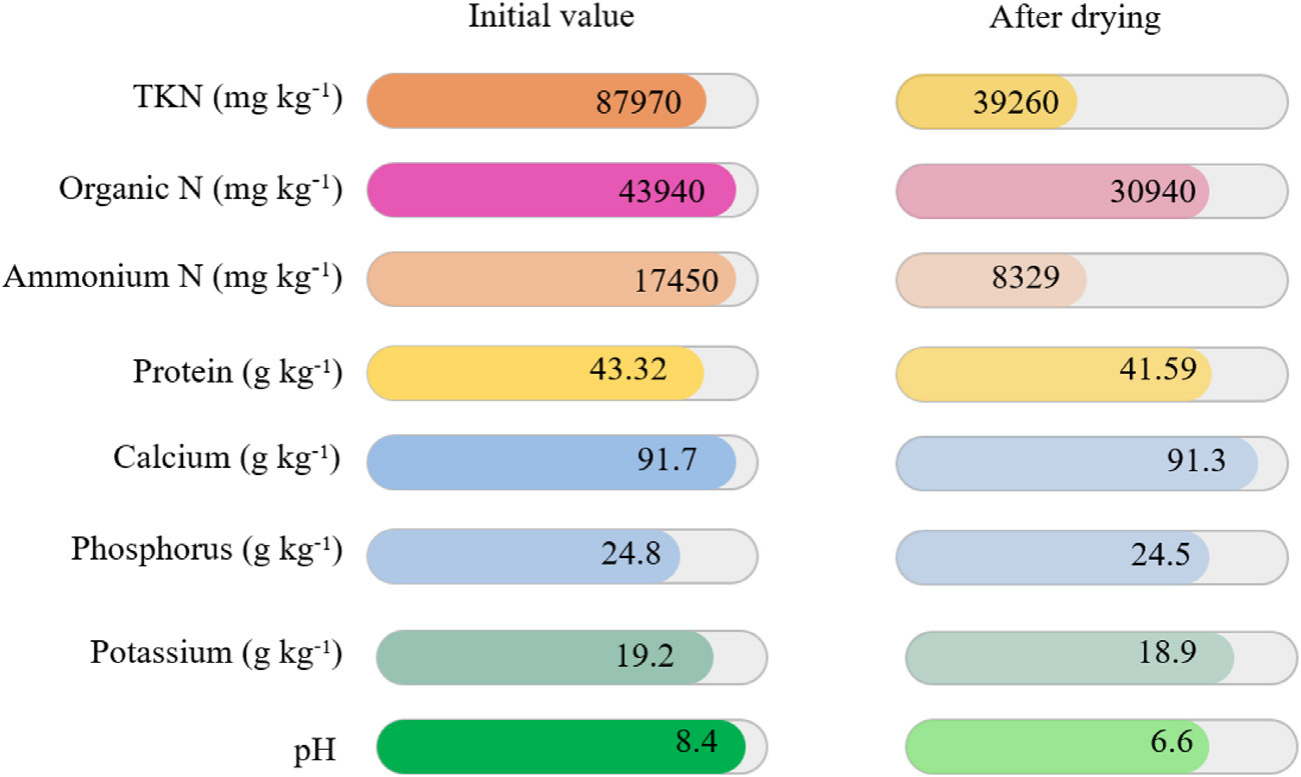
Laying hen manure characteristics before and after solar drying (Ghaly and MacDonald, 2013).

Ghaly and Alhattab (2013) investigated the effects of drying depth (1, 2, and 3 cm) and temperature (40, 55, and 60°C) on the nutritional profile of the dried laying hen manure in a solar dryer. Manure depth and drying temperature had no significant effect on the manure pH. Nevertheless, the release of ammonia during the drying process led to a decrease in manure pH from 8.4 to 6.6, which caused a reduction of nitrogen content from 4.58 to 2.07 and phosphorus content from 1.29 to 1.28. The potassium content was unchanged during the drying process. In general, solar drying of laying hen manure led to the reduction of organic nitrogen, total Kjeldahl nitrogen (TKN), ammonium nitrogen, pH, protein, calcium, phosphorus, and potassium. Figure 2 shows the reported values of these elements by Ghaly and Alhattab (2013) before and after drying (Ref). The researchers believed that the high loss in nitrogen content was due to the volatilization of ammonia during the solar drying process.

The effects of oven drying at 65°C, hot air drying at 22°C, and freeze drying on the phosphorus (P) content of laying hen manure were examined by Dail et al. (2007). They reported that among 3 drying methods, the water-soluble P fraction showed the most alteration. Phosphorus became more sensitive to heat applied in the drying process because drying increased the H_2_O-extractable phosphorus and decreased the NaHCO_3_-extractable phosphorus. It appears that the drying process causes the relatively stable phosphorus in laying hen manure to become more unstable or resistant. This bidirectional trend may partially explain the contradictory observations of the drying process reported by other researchers. Consequently, variations due to drying effects should be considered when evaluating laying hen manure for P availability or when comparing data where different drying approaches have been used.

This literature review shows that the elemental change of the poultry litter during the drying process has been well-attended by researchers. However, it is important to mention that despite the review of different reported research, no research has been reported for rotary dryers and belt dryers. The type of drying method can affect the content of poultry litter elements. Also, the type of poultry litter shows different behaviors in different drying methods. Poultry litter pH decreases in all drying methods. The optimal conditions for drying poultry litter are different according to its uses. Drying poultry litter at low temperatures and low air speeds, with the goal of using it as a protein source, can result in a decrease in protein content due to the extended drying time. However, this trend is reversed for laying hen manure. Drying poultry litter at low temperatures does not have a significant effect on changes in its nitrogen content, but drying at high temperatures can reduce the nitrogen content of poultry litter. Drying can lead to an increase in the content of Ca, P, K, Mg, Fe, Cu, Zn, Mn, etc., but it also leads to a decrease in water-soluble elements such as K and B.

## ENERGY CONSUMPTION DURING THE DRYING PROCESS

One of the most important indicators in evaluation of drying as a unit operation is energy consumption. However, researchers are still limited in choosing energy sources that reduce drying duration and energy consumption (EL-Mesery, 2022). This also applies to the poultry litter drying, as very little research has been reported on the energy consumption analysis of drying this manure. Here the reported studies are presented.

### Broiler Litter Energy Consumption

The effect of the air relative humidity, air temperature, air velocity, and depth of broiler litter on specific energy consumption during the hot air drying of the broiler litter was investigated by Khodadadi et al. (2023b). They reported that the minimum value for the specific energy consumption was 3.38 MJ kg^-1^. This optimal value was attained at an air relative humidity of 18%, an air velocity of 2 m s^-1^, an air temperature of 80°C, and a broiler litter depth of 2 cm. As the relative humidity of the air and air temperature increased, the specific energy consumption decreased. Also, at low temperatures, specific energy consumption increased. Reduction in the air temperature had a direct effect on the increase of specific energy consumption according to Eq. 2.

(2) $$\text{SE}=\;\left(\frac{t}{m_{v}}\right)\left[\frac{Q(c_{\text{pa}}+\;c_{\text{pv}}h_{a})}{V_{h}}\right]\left(T_{\text{in}}\;-\;T_{\text{am}}\right)$$

### Laying Hen Manure Energy Consumption

Energy consumption when the laying hen manure is dried by a hot air dryer was studied by Li et al. (2020b). Their results showed that energy consumption rose with the increase in the manure depth and reduced with the increase in the air velocity and temperature. Because with the increase in manure depth, the drying time and, consequently, the energy consumption also increase, and the drying time decreases as the air temperature rises. and as a result, the energy consumption is reduced. However, the most significant factor affecting the amount of energy consumption was the air temperature.

Li et al. (2020a) observed that raising the air temperature in a hot air dryer reduced energy consumption during the low-temperature drying process. A potential reason was that higher drying temperatures lead to shorter drying times and thus less energy consumption.

These literature survey reveals that not much research has been performed in this field. Consequently, it is recommended to carry out various investigations on the energy consumption for drying poultry litter in different dryers. Optimizing poultry litter drying in various dryers and analyzing its economic feasibility can aid researchers in selecting the best drying method.

## CHANGES IN MICROBIAL LOAD DURING THE DRYING PROCESS

There is a vast and diverse community of microorganisms in poultry litter. Gram-positive bacteria, including *clostridia/eubacteria bacilli/lactobacilli*, and actinomycetes, constitute almost 90% of the microbes in the poultry manure. Furthermore, up to 10 log cfu g^-1^ of bacteria have been found in the poultry litter. There are Various human pathogens in the poultry litter, such as *Campylobacter, Actinobacillus, Clostridium, Bordetella, Escherichia coli, Corynebacterium, Globicatella, Listeria mycobacterium, Staphylococcus, Salmonella*, and *Streptococcus* (Bolan et al., 2010). Currently, heat treatment is one of the most popular techniques to eliminate pathogens in animal waste (Thermal drying). The main factors affecting pathogen reduction through the drying process are temperature, duration of heating, and moisture content. However, some pathogens may have the potential to adapt to the hostile environment during heat treatment and protect them from subsequent high-temperature treatments (Singh and Jiang, 2012). Therefore, some existing guidelines for heat treatment of poultry litter may not be sufficient to remove some pathogens from this manure, and it is necessary to use other methods to reduce the microbial load of poultry litter, such as the use of ultraviolet (**UV**) sterilization. The use of UV radiation to inactivate microorganisms in animal manure has been proven to be an effective alternative (Singh et al., 2006). The previous studies reported that Treatment with UV is a common option for inactivating waterborne organisms such as E. coli (Romero-Martínez et al., 2023). The advantages of the UV treatment include the absence of chemicals that facilitates the treatment application, avoids the need for storing potentially hazardous materials, and limits the generation of disinfection by-products (Silva et al., 2013). Due to the importance of this issue, it is necessary to review the literature in this case as a guideline for future research.

### Broiler Litter Microbial Load

Biktasheva et al. (2019) compared 2 broiler litter drying methods (high-temperature drying at 250°C for 30 s and slow pyrolysis at 400°C for 2 h on the presence of *Listeria monocytogenes, Salmonella spp, E. coli, Mycobacterium paratuberculosis, Campylobacter jejuni, Enterococcus spp, Clostridium perfringens, Bacillus anthracis, Yersinia enterocolitica, Bacillus cereus, Bacillus thuringiensis*, and *Streptococcus dysgalactiae*. They reported that drying at high temperatures did not consequence in significant variations in the number of primary pathogenic microorganisms, while pyrolysis destroyed all pathogens. Therefore, regarding dried broiler litter characteristics, slow pyrolysis is a desirable method for heat treatment of the broiler litter.

The effectiveness of thermal treatment in inactivating *Salmonella* in broiler litter was assessed by Chen et al. (2015). Their results showed the effect of broiler litter moisture on the thermal inactivation of *Salmonella*. Higher initial moisture content in the broiler litter helped to kill *Salmonella* more quickly during wet heat treatment. In this regard, they stated that water molecules that have direct contact with proteins inside the cell could be an influential factor in microbial inactivation. In this investigation, no significant reduction in the *Salmonella* population was observed during the dry heat treatment. Consequently, a 2-step heating process including a wet heat treatment for 1 h at 65°C, and a consecutive dry heat treatment for 1 h at 85°C can reduce more than 5.5 log of *Salmonella* in the broiler litter with more than 40% moisture content.

Chen et al. (2013) investigated the effect of heat treatments of 70, 75, 80, 85, and 150°C on *Salmonella* counts of the broiler litter. By exposing the broiler litter to these temperatures, the *Salmonella* counts in the manure decreased, with the difference that the rate of decrease was higher at high temperatures. During thermal exposure, a discernible variation in heat resistance was seen among various *Salmonella* serotypes, since *S. Typhimurium* and *S. Senftenberg* showed higher levels of thermal resistance than *S. Heidelberg* and *S. Enteritidis*.

Kim et al. (2012) reported that by drying fresh broiler litter with a moisture content of 30%, 40%, and 50% in a conventional oven at 80°C for 44.1 to 63.0 min, a 7-log decrease of *Salmonella* species can be achieved. When they examined the effect of freshness of the broiler litter on heat-resistance of *Salmonella, Salmonella cells* exhibited a considerably longer survival rate in aged broiler litter compared to those in fresh broiler litter under all conditions.

According to Wilkinson et al. (2011), the total number of *Salmonella* and *E. Coli* decreased by above 99% in the broiler litter exposed to either 55°C or 65°C for 1 h. The fastest reduction in bacteria in *Salmonella* and *E. coli* counts occurred at higher temperatures, nonetheless 65°C was not more effective than 55°C. After a 1-h period of being subjected to these temperatures, over 99% of the *E. Coli* counts were reduced, and *Salmonella* was eliminated entirely.

Nguyen et al. (2022) studied the deactivation of airborne *E. coli* by ultraviolet (UV) radiation from poultry dust particles. At various contact times, the deactivation rate of airborne *E. Coli* was examined. (0.23 to 5.62 s) with the help of a vacuum pump and different levels of UV radiation (1707 µW cm^-2^ and 3422 µW cm^-2^). Their findings indicated that the deactivation rates ranged from 99.87% to 99.95% at 5.62 s contact time with 1707 µW cm^-2^ and 3422 µW cm^-2^ of UV radiation and from 72.90% to 86.60% at 0.23 s contact time with 1707 µW cm^-2^ and 3422 µW cm^-2^ of UV radiation.

### Laying Hen Manure Microbial Load

The effect of air velocity (1, 1.5, and 2 m s^-1^), air temperature (70, 80, and 90°C), and manure depth (5, 10, and 15 mm) on changes in *Salmonella* counts of the laying hen manure during dying in a hot air dryer was investigated by Khademi et al. (2024). They reported that no bacteria were detected at all velocities and manure depths at temperatures of 90°C. At 70 and 80°C, a small number of bacteria were observed, but their number was almost the same at all velocities and thickness of the layers.

Li et al. (2020a) reported no difference between the amounts of *E. coli* or total bacteria in the laying hen manure dried at 15, 25, and 35°C and drying at low temperatures caused poor destruction of bacteria in the laying hen manure.

Mahmoud (2017) studied the effects of manure depth (1, 2, and 3 cm) and drying temperature (40, 50, and 60°C) on the microbial load of the laying hen manure. The number of bacteria, *E. Coli*, and *yeast-mold* decreased by 65.62%-99.83%, 99.97%, and 74.07%-99.63%, respectively. They also reported that *Salmonella* was destroyed entirely at temperatures greater than 50°C.

Ghaly and MacDonald (2012b) investigated the changes in microbial counts of the laying hen manure during the drying in a solar dryer. The results showed that there are a large number of bacteria (477 × 10^7^ cells/g manure) and *mold* and *yeast* cells (2,700 cells g^-1^ manure) within the raw manure. The process of drying laying hen manure with a depth of 3 cm and at a temperature of 60°C reduced the number of microorganisms by 99.87% (from 477 × 10^7^ to 620 × 10^4^ cells g^-1^ manure), *mold* and *yeast* cells by 99.63% (from 2,700 to <10 cells g^-1^) and reduced *E. coli* by 99.56% (from 2,290 × 10^4^ to <10 × 10^4^ cells g^-1^). *Salmonella* was present in the raw laying hen manure to some extent, but it was not detected in chicken manure after drying at a temperature of 60°C and a depth of 3 cm. They stated that metabolic activity, survival, and growth of an organism are affected by temperature, and heat-killing action is a temperature and time-dependent process. Practical procedures in which heat is used to kill an organism are divided to: (1) moist heat and (2) dry heat. There is a significant difference between these 2 methods in the efficiency of killing microorganisms. Moist heat kills microorganisms by denaturing cellular proteins, which is facilitated by the presence of moisture. Conversely, dry heat dehydrates the cell and kills microorganisms by oxidizing intracellular components. Studies on heat inactivation of pathogens have shown that dry heat requires much higher temperatures than moist heat (Kim et al., 2012).

As explained, various researches have been conducted on the changes in the microbial load of the poultry litter during the drying process. Still, these researches do not cover all the dryers used to dry poultry litter. This indicates that changes in the microbial load of the poultry litter still requires much more extensive research. However, some results can be summarized as follows: Temperature exerts a significant influence on the metabolic activity, survival, and growth of organisms. The thermal inactivation of microorganisms is a process that is directly influenced by both temperature and exposure time. The decrease rate of microbial load is higher at high temperatures. Drying poultry litter at temperatures below 35°C does not have a significant effect on the amount of *E. coli* or total bacteria. But *salmonella* is completely destroyed at a temperature above 50°C. *Salmonella* bacteria in aged broiler litter exhibit a significantly higher survival rate than those found in fresh broiler litter. The heat resistance of *Salmonella* serotypes varies significantly (*S. Typhimurium* and *S. Senftenberg* have higher levels of heat resistance than *S. Heidelberg* and *S. Enteritidis*). High initial moisture content in poultry litter enhances the effectiveness of wet heat treatment in killing *Salmonella*. Conversely, dry heat treatment has a negligible impact on *Salmonella* populations.

## AMMONIA EMISSION DURING THE DRYING PROCESS

Nitrogenous compounds in the poultry litter such as, ammonium, urea, and undigested proteins are possible sources for ammonia production, provided that the pH, temperature, and moisture content are suitable for microbial activity (Castesana et al., 2018). In oxygen presence, the nitrogenous compounds are reformed into carbon dioxide, nitrate, sulfate, and water. Under aerobic conditions, amino acids, peptides, and proteins are transformed to soluble ammonia by heterotrophic bacteria, and then in turns into ammonium by captivating H^+^ from water. Lastly, based on the temperature and pH of the ammonium source, ${\text{NH}}_{4}^{+}$ is converted to NH_3_ (Adeniyi et al., 2023). Therefore, the amount of nitrogen in the poultry litter is directly related to the amount of moisture. An increase in temperature causes more ammonia to dissolve in water and turn into free NH_3_, which can enter the atmosphere by evaporating the water inside the poultry litter. The results of some researches have confirmed that ammonia emission from dry manure is significantly less compared to the fresh manure (Rosa et al., 2020). Therefore, it is necessary to investigate the effect of various drying methods on releasing ammonia from the poultry litter. In this section, the results of some researchers regarding the ammonia emission from the poultry litter are given and discussed.

### Ammonia Emission from Broiler Litter

Khodadadi et al. (2023a) investigated broiler litter drying by a hot air dryer and its effect on ammonium nitrogen changes to give an overview of the ammonia emission. In this research, the effect of the air velocity (2–3 m s^-1^), air temperature (60–80°C), manure depth (2-4 cm), and air relative humidity (8%–18%) were analyzed on ammonia emission. Air velocity, air temperature, manure depth, and air relative humidity positively correlated with ammonia emission. By increasing the depth of the manure or decreasing the air velocity, the drying duration increased and led to more decomposition of organic nitrogen into ammonium nitrogen through aerobic fermentation, and consequently more ammonia released. In high air humidity values, ammonia emission increased with increasing air temperature.

Rubežius et al. (2020) observed that the release of ammonia from the broiler litter was very sensitive to moisture content. Their findings showed that the interaction effects of different factors for optimal response in ammonia emission during the drying process of the broiler litter was not considered.

Hou et al. (2017) reported that the ammonia emission from the dried broiler litter and laying hen manure was considerably lower than the fresh manure.

**Figure 3:**
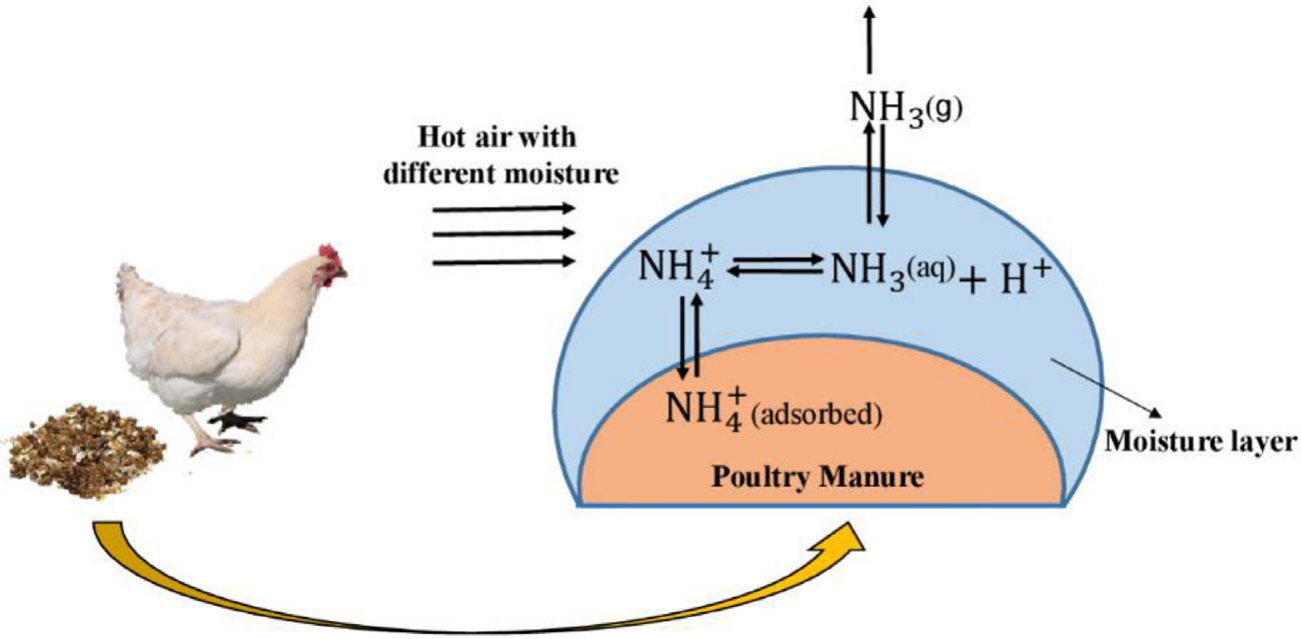
The process of ammonia release from poultry litter (Liu et al., 2007).

Liu et al. (2007) designed a dynamic flow-through chamber apparatus to measure ammonia emissions from the broiler litter. They observed that the ammonia emission from the broiler litter was very sensitive to its moisture content. Adding water to the broiler litter increased the total ammonia nitrogen content and could potentially increase ammonia emissions. They also reported that temperature was an important variable in ammonia emission processes from the broiler litter. Air temperature affects the convective coefficient of mass transfer. Also, litter temperature affects the dissociation constant, Henry's constant, as well as the diffusion and ammonia generation in the broiler litter. Therefore, temperature changes can significantly affect the flow of ammonia. They have summarized the process of ammonia release from poultry litter in Figure 3.

### Ammonia Emission from Laying Hen Manure

Li et al. (2020a) studied the effect of the drying temperature on ammonia emission from the laying hen manure. They reported that the NH_4_-N content of the manure after 8 h drying at 15°C and 25°C was not significantly different. However, the temperature of 35°C significantly reduced the NH_4_-N content of the manure, which indicates the higher release of ammonia at this temperature. When the drying process continued until the end of drying, the NH_4_-N content of the dried laying hen manure varied significantly at different temperatures. They explained the reason as follows: NH_4_-N in manure mainly comes from the breakdown of uric acid and undigested proteins, which is highly soluble in water. Raw laying hen manure has the appropriate moisture content to hold NH_4_-N, which is produced through the process of ammonification. As the temperature increases, the dissociation constant increases, and the driving force of heat transfer increases. This leads to an increase in the rate of water evaporation, so the higher the temperature, the faster and more is the loss of NH_4_-N and, and as a result the release of ammonia.

The impact of tunnel drying on ammonia release from laying hen manure was examined by Rosa et al. (2020). They reported that the average ammonia emission was 209.3 mg NH_3_ d^-1^ hen^-1^, and the release of ammonia influenced by the atmospheric temperature, air relative humidity, and the dry matter content of the manure. The decrease in NH_3_ concentration was in accordance with the moisture losses of manure, which confirmed that NH_3_ production reduced from 60% of DM content. Also, the movement of the belt was recognized as an important factor in releasing ammonia.

Tong et al. (2020) investigated on the ammonia emission from the laying hen manure during drying by a commercial manure belt (air temperatures of 20–33.5°C) and air velocities of 0.25–1.17 m s^-1^). They reported that drying at higher temperatures led to an apparent decrease in NH_3_ emission.

Winkel et al. (2017) evaluated the effect of tunnel drying on ammonia emission from the laying hen manure. Their results indicated that upon passing the tunnel dryer, the ammonia concentration in the drying air increased considerably from an average of 5.5 ppm upstream to an average of 13.9 ppm downstream the manure drying tunnel. Increased concentration of ammonia was significantly higher for belt-type manure drying tunnel than for plate-type manure drying tunnel, maybe due to quicker drying.

Ghaly and MacDonald (2013) reported that drying laying hen manure resulted in a significant nitrogen loss through ammonia losses. Ndegwa et al. (2008) stated that in laying hen facilities, drying manure on a belt reduces ammonia emissions by 60% or more.

Existing research indicates that the moisture content of poultry litter has a profound effect on ammonia emissions. Increasing the moisture content of poultry litter through the addition of water resulted in a higher total ammonia nitrogen concentration, which could potentially lead to increased ammonia emissions. During the drying process, air velocity, air temperature, manure depth, and air relative humidity positively correlate with ammonia emissions. Drying poultry litter at high temperatures leads to a significant reduction in ammonia (NH_3_) emissions. Ammonia emissions from dried broiler litter and laying hen manure are significantly lower compared to their fresh counterparts.

## CONCLUSIONS AND RECOMMENDATIONS FOR FUTURE PROSPECTS

This paper reviews and discusses different methods used for drying broiler litter and laying hen manure and the effect of each method on the key drying process indicators (drying kinetics, elemental content, energy consumption, microbial load, and ammonia emission). The literature review showed that in addition to the effective parameters in the drying process in each type of dryer, the drying method also affects the investigated indicators. The focus of the most research has been on the dryers that use hot air as the drying agent. This may be because the researchers believe that hot air has a more pronounced effect on the drying rate of the poultry litter or that high temperatures have a more significant effect on eliminating or reducing the microbial load of the manure. However, the heat treatment method may also affect the drying process, and consequently the content of elements, drying kinetics, energy consumption, and even the release of ammonia, which shows the importance of using different methods for drying manure. Also, there is a lack of comprehensive research on poultry litter drying with modern drying methods (ultrasound, microwave, infrared radiation, and freeze dryers). Therefore, more research is still needed to investigate the application of drying with different dryers. Hybrid methods, which combine hot air-drying agents with modern drying methods, may present new opportunities.

## DISCLOSURES

The authors declare that they have no known competing financial interests or personal relationships that could have appeared to influence the work reported in this paper.
